# Rev-erbα agonist SR9009 protects against cerebral ischemic injury through mechanisms involving Nrf2 pathway

**DOI:** 10.3389/fphar.2023.1102567

**Published:** 2023-03-31

**Authors:** Mingyue Sheng, Xun Chen, Yan Yu, Qi Wu, Junping Kou, Gangling Chen

**Affiliations:** ^1^ Department of Pharmacology of Chinese Materia Medica, School of Traditional Chinese Pharmacy, China Pharmaceutical University, Nanjing, China; ^2^ State Key Laboratory of Natural Products, Jiangsu Key Laboratory of TCM Evaluation and Translational Research, Department of Complex Prescription of TCM, China Pharmaceutical University, Nanjing, China; ^3^ State Key Laboratory of Natural Medicines, Research Department of Pharmacognosy, School of Traditional Chinese Pharmacy, China Pharmaceutical University, Nanjing, China

**Keywords:** cerebral ischemia, SR9009, REV-erbα, Nrf2, circadian rhythm, circadian gene

## Abstract

**Backgrounds:** The circadian clock protein Rev-erbα is a crucial regulator of circadian rhythms that affects multiple molecular, cellular, and physiology pathways that control susceptibility, injury, and recovery in the neurological disorders. Emerging evidence suggest that Rev-erbα plays a key role in the inflammation and oxidative stress, two pivotal mechanisms in the pathogenesis, progression, and recovery process of ischemic stroke. However, it remains inconclusive whether Rev-erbα activation is protective against ischemic brain damage. Nuclear factor erythroid 2-related factor 2 (Nrf2) pathway, a master regulator of inflammatory and oxidative responses. Our study aimed to determine whether pharmacological activation of Rev-erbα by SR9009 protects against acute ischemic brain damage partly *via* Nrf2 pathway.

**Methods:** Adult mice were pretreated with SR9009 or Nrf2 inhibitor all-trans-retinoic acid (ATRA) for 3 days prior to Sham or middle cerebral artery occlusion (MCAO) operation. After ischemia for 1 h and reperfusion for 24 h, the neurological function and cerebral infarction volume were determined, superoxide dismutase (SOD) activity, malondialdehyde (MDA) content and glutathione peroxidase (GSH-PX) activity in serum were detected by kit. The mRNA and/or protein level of tumor necrosis factor-α (TNF-α), interleukin-1β (IL-1β), inducible nitric oxide synthase (iNOS), Period (Per)1, Brain and muscle arnt-like1 (Bmal1), Circadian locomotor output cycles kaput (Clock), Rev-erbα, Nrf2, heme oxygenase-1 (HO-1) and quinone oxidoreductase 1 (NQO1) in cerebral cortex were detected by q-PCR and Western blot.

**Results:** We confirmed that SR9009 activated Rev-erbα gene in the cerebral cortex under basal condition. At 24 h after reperfusion, SR9009 ameliorated acute neurological deficits, reduced infarct volume. Meanwhile, the inflammatory TNF-α, IL-1β, iNOS and MDA content levels were significant decreased, SOD and GSH-PX activity were obviously increased, which were markedly blunted (or abolished) by ATRA. SR9009 enhanced the induction of Nrf2 and its downstream target genes HO-1 and NQO1 after ischemic insult. In addition, we found that SR9009 restored Rev-erbα, Bmal1, Clock, Per1 genes expression in the cerebral cortex under ischemic condition.

**Conclusion:** Taken together, Rev-erbα activation by SR9009 protects against ischemic stroke damage, at least, partly through Nrf2 pathway.

## Introduction

Ischemic stroke is one of the leading causes of mortality and disability worldwide (Virani et al., 2020). Inflammation (Orellana-Urzúa et al., 2020) and oxidative stress (Franke et al., 2020) have been widely known as two pivotal mechanisms for multiple neurological disorders, which are particularly important in the development and progression of ischemic pathology. Therefore, targeting these two mechanisms provides a promising new avenue to prevent and treat stroke. The nuclear factor erythroid 2-related factor 2 (Nrf2) has been shown to be a master regulator of inflammation and oxidative stress through diverse cytoprotective and detoxification genes like heme oxygenase-1 (HO-1) and quinone oxidoreductase 1 (NQO1), inducing various endogenous neuroprotective processes (Ya et al., 2018; Liu et al., 2020). In recent years, many therapeutic targets for pharmacological intervention have been identified to regulate endogenous neuroprotective mechanisms, which protect the brain from ischemic damage and facilitate its recovery.

Circadian rhythms, linked to various physiological processes, including sleep/wakefulness (Patke et al., 2020), metabolism (Panda, 2016), hormone secretion and neurobehavioral processes (Hanifin et al., 2020) are circa-24-h oscillations in biological processes (Aschoff, 1965). Most recent studies indicate that the disruptions of the circadian rhythms increase human susceptibility to ischemic stroke (Ramsey et al., 2020). The core of clock network is composed of circadian genes Brain and muscle arnt-like1 (Bmal1), Circadian locomotor output cycles kaput (Clock), Cryptochrome (Cry)1/2, Period (Per)1/2, Rev-erbα, etc (Mohawk et al., 2012). Rev-erbα (also known as NR1D1) is a Rev-erb family member and a nuclear hormone receptor (Everett and Lazar, 2014), which is dominant involved in circadian regulation (Janich et al., 2015). Rev-erbα is a major transcriptional silencer and a heme-responsive nuclear receptor that can be combined with small molecule agonists and antagonists (Solt et al., 2012). Recent findings showed that Rev-erbα is crucial in the regulation of inflammation (Griffin et al., 2019). It was shown that Rev-erbα activation by SR9009, the synthetic ligand for Rev-erbs, inhibited LPS-stimulated transcription of inflammatory factors IL-1β, IL-6, MMP-9 and Ccl2 in astrocytes (Morioka et al., 2019). SR9009 significantly attenuated hepatic damage and inflammatory responses (Lin et al., 2020a). SR9009 administration in mice at 1 day after myocardial ischemic-reperfusion prevents the heart failure by targeting the cardiac inflammasome (Reitz et al., 2019). However, the effect of Rev-erbα activation by SR9009 in ischemic stroke has not yet been reported.

Given the background above, in the present study, we aimed to elucidate whether Rev-erbα activation by SR9009 protects against neurological deficits and brain damage in a transient focal cerebral ischemic mouse model and whether the underlying mechanism involves Nrf2 pathways. These findings could contribute to clarifying the role of Rev-erbα activation in ischemic stroke.

## Materials and methods

### Animals and ethics statement

Male adult C57BL/6J mice (Qinglongshan, Nanjing, China) weighing 20–25 g were used in this research. Mice were housed in a temperature (25°C ± 1°C) and 12 h light/dark cycle-controlled room (light: 06:00, Zeitgeber time [ZT]0, dark: 18:00, ZT12) for 2 weeks (Fredrich et al., 2017). Food and water were available *ad libitum*. All animal experimental protocols and animal handling procedures were conducted in accordance with the Animal Ethics Committee of China Pharmaceutical University. All efforts were made to minimize animal suffering.

### Experimental transient cerebral ischemia model

Cerebral ischemia was induced by transient focal middle cerebral artery occlusion (MCAO) as described previously (Chen et al., 2020). Animals were deeply anesthetized with pentobarbital (Shanghai Civi Chemical Technology, Shanghai, China) during surgery. Focal cerebral ischemia was induced by a 6–0 nylon monofilament suture, blunted at the tip. The suture was inserted 9-10 mm into the internal carotid to occlude the origin of the MCA. An hour later, reperfusion was initiated by withdrawing the monofilament. Body temperature was maintained at 37°C by a heat pad during surgery. The sham-operated (Sham) mice were subjected to the same surgery procedure, except that the MCA was not occluded. All MCAO and sham surgeries were performed at ZT0.

### SR9009 administration

SR9009 is the synthetic ligand for Rev-erbs, designed based on the chemical structure of GSK4112 (the first synthetic ligand for Rev-erbs) (Solt et al., 2012). Either SR9009 (#orb363935, Biorbyt, Cambridge, England) or the Nrf2 inhibitor all-trans-retinoic acid (ATRA, #R106320, Aladdin, Shanghai, China) was dissolved in DMSO: Corn oil (5: 95). In experiment 1, mice were randomly divided into two sham groups: vehicle and SR9009 groups (*n* = 4 for each group). SR9009 was injected intraperitoneally at doses of 50 mg/kg at ZT6 for three consecutive days prior to MCAO (Yuan et al., 2019). Mice received sham-surgery on forth day. In experiment 2, mice were randomly divided into five groups: Sham, MCAO, SR9009, ATRA and SR9009 + ATRA groups (*n* = 8 for each group). ATRA was administrated intraperitoneally at doses of 10 mg/kg at ZT8 for three consecutive days. Mice in Sham and MCAO groups received equal volumes of vehicle at ZT6 for 3 days. Except for the Sham group, mice underwent MCAO surgery on the fourth day (Figure 2A).

## Behavioral testing

### Neurological deficit score

The overall neurological deficits were evaluated by neurological deficit scoring at 24 h after MCAO (Chen et al., 2020). The mice were placed on the horizontal and wide desktop. Six parameters: body symmetry, gait, climbing, circling behavior, front-limb symmetry, and compulsory circling were employed to observe the physical ability of mice.

### Rotarod test

The motor coordination function was assessed using an accelerating rotarod apparatus (Chengdu Taimeng Software Co., Ltd., Shenzhen, China) (Sunyer et al., 2007). Mice were trained on the perpendicular to the rod axis, with the head facing opposite direction of the experimenter for three consecutive trials at a slow rotational speed (10 rpm/min) for 5 min to adapt to the rod on the day before drug administration. At 24 h after surgery, the mice were tested with an accelerating rotational speed (from 4 to 40 rpm in 5 min). The latency to the first fall off the rod was recorded. Each mouse performed 3 trials with 15 min intervals. The average values were used for the final analyses.

### Open field test

The spontaneous locomotor activity was assessed by an open field paradigm with an automated tracking system (Chengdu Taimeng Software Co., Ltd., Shenzhen, China). Spontaneous locomotor activity data included the parameters activity number that indicates the travel distance and rearing times that indicate vertical exploratory preference. Each mouse was placed in the groove of the autonomous activity meter for 5 min. The chamber was cleaned with 70% ethanol and air dried between tests (Kim et al., 2020).

### Measurements of infarct volume

The infarct volume was assessed by 2, 3, 5-triphenyltetrazolium chloride (TTC) (17779, Sigma, Missouri, United States) staining at 24 h after reperfusion (Chen et al., 2020). Mice were sacrificed under anesthesia after neurological examination. The frozen brains were sliced into consecutive 1 mm coronal sections and immersed in 1% (w/v) TTC solution for 10 min at 37°C. Normal tissue was stained in red, while the infarct area showed pale gray. Image J software (NIH Image, National Institutes of Health, Bethesda, MD, United States) was applied to measure the infarct size. The percentage of the corrected infarct volume was calculated as: [volume of contralateral hemisphere—(volume of ipsilateral hemisphere–volume of infarct)/volume of contralateral hemisphere] *100.

### Determinations of SOD, GSH, MDA

Mice were anesthetized at 24 h after reperfusion, and whole blood samples were collected and stored at room temperature for 30 min, then centrifuged at 1,000 *g* for 30 min. The level of oxidative stress markers superoxide dismutase (SOD), glutathione peroxidase (GSH-PX) activity and malondialdehyde (MDA) content in serum were detected using SOD kit (A001-3), MDA kit (A003-1), GSH kit (A005) according to manufacturer’s instructions (Nanjing Jiancheng Bioengineering Institute, Nanjing, China).

### q-PCR

The total RNA for the penumbra of ischemic cortex was extracted using TRIzol reagent (R0016, Beyotime, Shanghai, China). Nano (NANO-400, Shanghai, China) was used to quantify the concentration of RNA sample at 260/280 nm, and the samples with 1.8 < A260/280 < 2.0 were selected for further experiment to ensure RNA quality. cDNA was transcribed using HiScript II Q RT SuperMix for qPCR (+*g* DNA wiper) Kit (R223-01, Vazyme, Nanjing, China) to transcribe cDNA from 1.0 μg RNA. Use ABI QuantStudio 3 real-time PCR detection system (Thermo Fisher Scientific, United States) and ChamQ SYBR qPCR Master Mix (Low ROX Premixed) (Q331-02, Vazyme, Nanjing, China) for q-PCR analysis. The thermal cycling parameters were as follows: after preincubation for 30 s at 94°C, 40 cycles of amplification (95°C for 10 s, 60°C for 1 min) were performed. GAPDH was used as an internal control. The expression level of target gene was normalized to the expression level of GAPDH using the 2^−ΔΔCt^ method (Ct = Threshold Cycle). The primer sequences were shown in Table 1.

**TABLE 1:** Primer sequences for qPCR.

Primer name	Forward primer (5′–3′)	Reverse primer (5′–3′)
Per1	CAG​CCG​TGC​TGC​CTA​CTC​ATT	AGA​GGC​AGT​TGG​TGT​GTG​TC
Clock	CCT​ATC​CTA​CCT​TCG​CCA​CAC​A	TCC​CGT​GGA​GCA​ACC​TAG​AT
Bmal1	CCA​AGA​AAG​TAT​GGA​CAC​AGC​AAA	GCA​TTC​TTG​ATC​CTT​CCT​TGG​T
Rev-erbα	CCC​TGG​ACT​CCA​ATA​ACA​ACA​CA	GCC​ATT​GGA​GCT​GTC​ACT​GTA​G
Nrf2	ATG​ATG​GAC​TTG​GAG​TTG​CC	TCC​TGT​TCC​TTC​TGG​AGT​TG
HO-1	ATG​TGG​CCC​TGG​AGG​AGG​AGA	CGC​TGC​ATG​GCT​GGT​GTG​TAG
NQO1	TTT​AGG​GTC​GTC​TTG​GCA​AC	GTC​TTC​TCT​GAA​TGG​GCC​AG
TNF-α	CCT​GTA​GCC​CAC​GTC​GTA​G	GTC​TTC​TCT​GAA​TGG​GCC​AG
IL-1β	GAA​ATG​CCA​CCT​TTT​GAC​AGT​G	TGG​ATG​CTC​TCA​TCA​GGA​CAG
iNOS	CGG​ATA​GGC​AGA​GAT​TGG​AG	GTG​GGG​TTG​TTG​CTG​AAC​TT
GAPDH	AAA​TGG​TGA​AGG​TCG​GTG​TGA​AC	CAA​CAA​TCT​CCA​CTT​TGC​CAC​TG

### Western blot

Protein extractions in the penumbra of ischemic cortex were obtained using a total protein extraction kit (FD009, FDbio, Hangzhou, China) following the manufacturer’s protocols. Then the BCA Protein Assay Kit (AR1189, BOSTER, California, United States) was used to determine the protein concentration. Loaded an equal amount of protein sample on sodium dodecyl sulfate-polyacrylamide gel electrophoresis (SDS-PAGE), and then electrotransferred onto PVDF membrane. The membrane was blocked with 5% skimmed milk powder in Tris buffered saline and incubated with anti-PER1 (A8449, ABclonal, Boston, United States, 1:1,000), anti-BMAL1 (A17334, ABclonal, Boston, United States, 1:850), anti-CLOCK (A5633, ABclonal, Boston, United States, 1:1,000), anti-NR1D1 (BM5531, BOSTER, California, United States, 1:350), anti-NRF2 (PB9290, BOSTER, California, United States, 1:1,000), anti-HO-1 (A1346, ABclonal, Boston, United States, 1:1,000), anti-NQO1 (A0047, ABclonal, Boston, United States, 1:1,000) overnight at 4°C. The membrane was then incubated with the HRP-conjugated Streptavidin (BS10044, Bioworld, Minnesota, United States) for 2 h at 37°C and detected using an enhanced chemiluminescence (E412-01, Vazyme, Nanjing, China). Image analysis software was used to analyze the optical density of the protein bands. These values were normalized to the GAPDH content and expressed as relative intensity.

### Statistical analysis

All data was statistically analyzed by using GraphPad Prism 8.0 software (GraphPad software, Inc., La Jolla, CA, United States). Results were expressed as mean ± SEM. Student’s *t*-test was used for the comparison between the two groups. One-way ANOVA analysis was used for the comparison of three or more groups, followed by using Dunnett’s test, *p* < 0.05 was considered to be statistically significant.

## Results

### SR9009 activates Rev-erbα in cerebral cortex of mice

**FIGURE 1:**
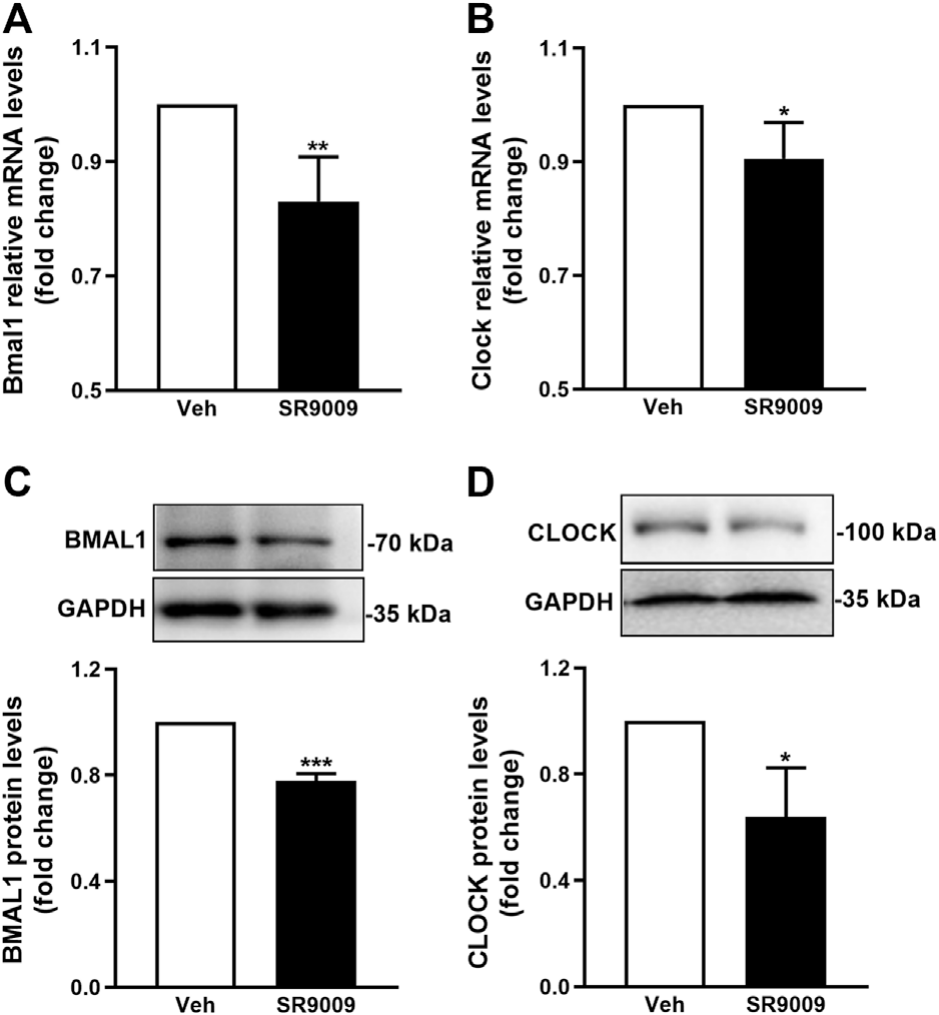
Pharmacological activation of Rev-erbα by SR9009. Male mice (C57BL/6J) received a once-daily injection of either SR9009 (50 mg/kg; i.p.) or vehicle during the morning hours (12:00 a.m., ZT6) for 3 days prior to sham-surgery. The activation of Rev-erbα in the cerebral cortex was examined 24 h after sham-surgery, indicated by the mRNA **(A,B)** and protein **(C,D)** expression levels of Bmal1 and Clock. Compared to vehicle controls, Real-time PCR and Western blot analysis revealed that SR9009 induced dramatic reduction in both mRNA and protein levels of Bmal1 and Clock. Data are expressed as mean ± SEM, *n* = 3. ^*^ *p* < 0.05, ^**^ *p* < 0.01, ^***^ *p* < 0.001.

To confirm whether SR9009 activates Rev-erbα in the brain, adult naive C57BL/6J mice received treatment with either SR9009 or vehicle for 3 days prior to sham surgery, and the Rev-erbα activation was estimated by western blot and q-PCR at 24 h after sham-surgery. The activation of Rev-erbα was reflected by Bmal1 and Clock protein expression levels which is controlled in a Rev-erb–dependent manner (Mohawk et al., 2012). As shown in Figure 1, western blot and q-PCR results revealed that SR9009 markedly decreased the genes and proteins levels of Bmal1 and Clock compared with vehicle controls (*p* < 0.001). Together, these data suggest the activation of Rev-erbα in the cerebral cortex of mice by SR9009 treatment.

### Rev-erbα activation by SR9009 ameliorates neurological deficits after ischemic stroke

**FIGURE 2:**
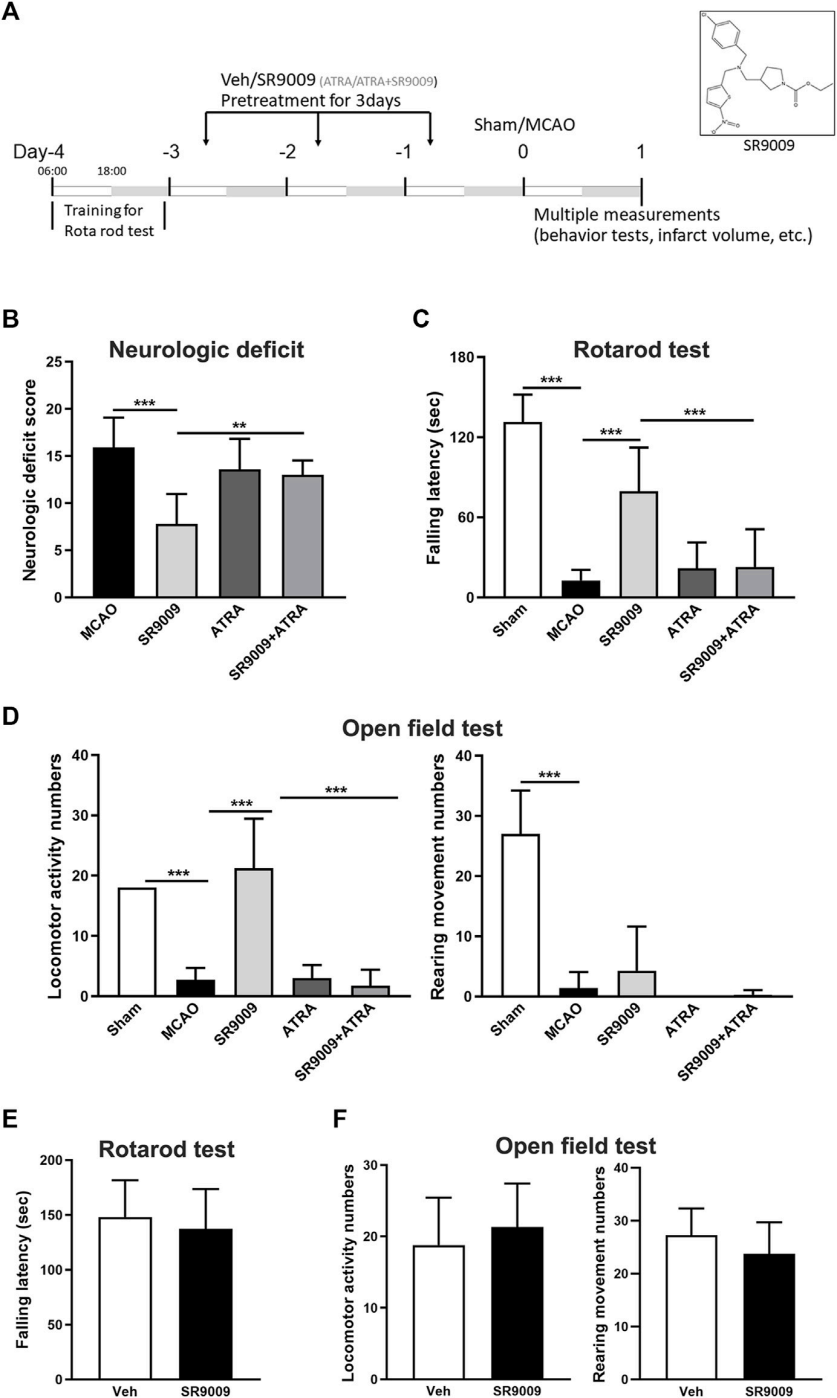
The Rev-erbα activation by SR9009 attenuates ischemia-induced acute neurological deficits. **(A)** Experimental design. 6:00 a.m. (ZT0) as the beginning of the light phase, and 18:00 a.m. (ZT12) as the start of the dark phase. Male mice (C57BL/6J) were pre-treated with SR9009 (50 mg/kg, i.p.), ATRA (10 mg/kg, i.p.), ATRA + SR9009 (i.p.), or vehicle for 3 days and then subjected to MCAO surgery. At 24 h after MCAO, SR9009 significantly reduced the neurological deficits score **(B)**, increased the fall latency in the rotarod test **(C)**, and reduced the locomotor activity numbers and rearing times in the open field paradigm **(D)**. Nrf2 inhibitor ATRA eliminated the protective effect of SR9009. No significant differences were detected between Vehicle group and Vehicle add SR9009 groups in the rotarod and the open field tests **(E,F)**. Data are expressed as mean ± SEM, *n* = 6–8. ^**^ *p* < 0.01, ^***^ *p* < 0.001.

To test whether SR9009 is protective against ischemic injury, multiple neurobehavioral and histological outcomes were evaluated at 24 h after MCAO. It is shown that SR9009 significantly reduced the ischemia-induced neurological deficits, indicated by the reduced neurological deficit score (*p* < 0.001) (Figure 2B). Meanwhile, the motor function decline following MCAO was ameliorated by SR9009, which was revealed by the latency to fall in the rotarod test (Figure 2C). SR9009 also exhibited obvious protective effect in the ischemia-induced reduction of spontaneous locomotor activity, reflected by the locomotor activity number and the rearing times in the open field test (Figure 2D). Interestingly, the Nrf2 inhibitor ATRA treatment exacerbated the neurological deficits in the behavior tests above. In contrast, such protective effects of SR9009 in the functional marker above were significantly reduced when administered with the Nrf2 inhibitor ARTA, suggesting the underlying mechanisms might involve Nrf2 pathway. We also further determined whether SR9009 could affect spontaneous locomotor activity and locomotor activity under basal condition. As expected, no significant difference was observed between groups in the rotarod (Figure 2E) and the open field tests (Figure 2F). Together, these findings support the functional protection of SR9009 on ischemic stroke injury.

### REV-erbα activation by SR9009 reduces the infarct volume after ischemic stroke

**FIGURE 3:**
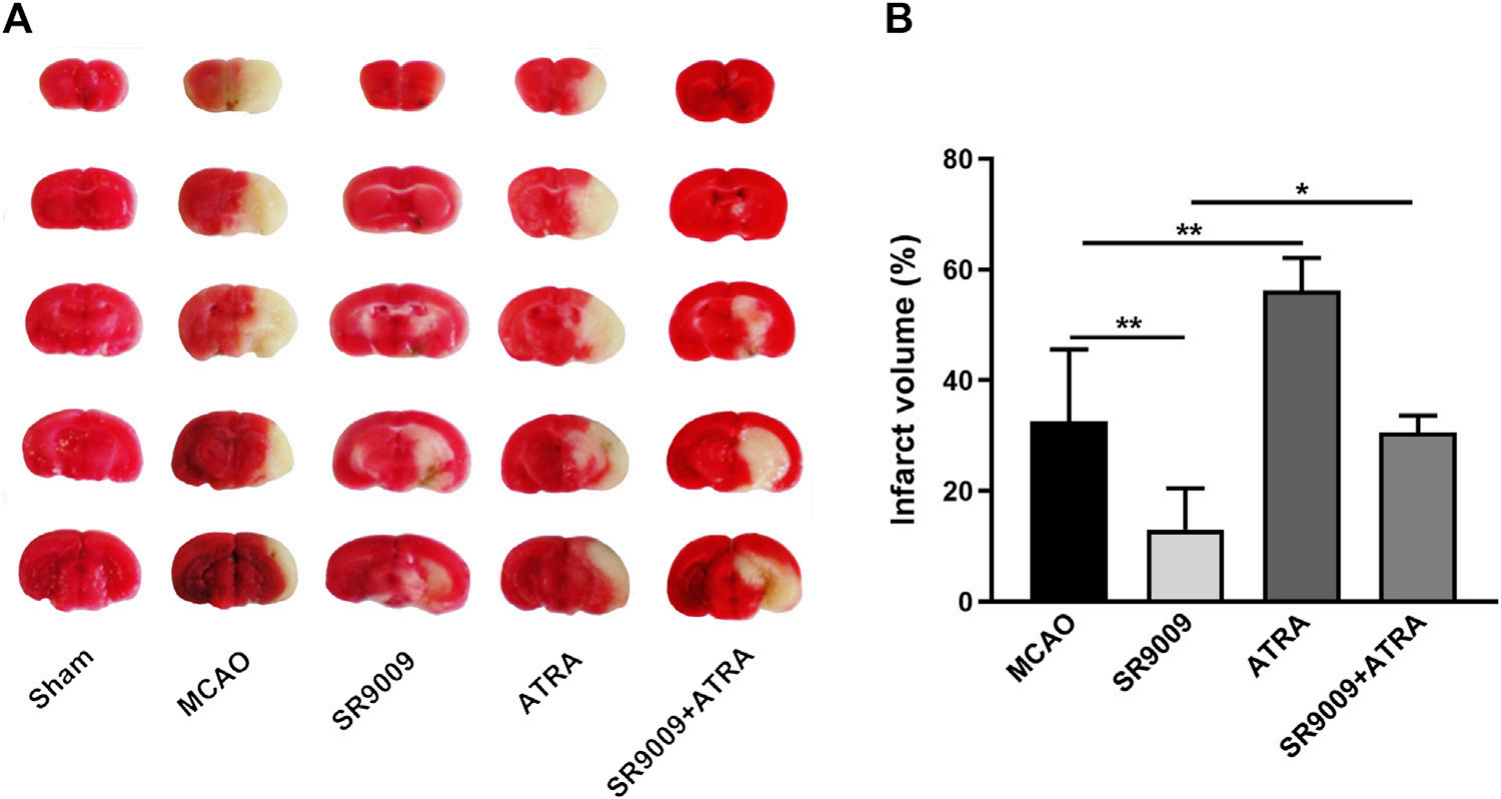
The Rev-erbα activation by SR9009 reduces the infarct volume after ischemic stroke. The effect of SR9009 pretreatment on infarct volume 24 h after ischemic stroke was determined by TTC staining. The representative photograph **(A)** and quantification **(B)** of infarct volume in serial coronal sections showed that SR9009 markedly reduced the infarct volume compared with MCAO controls. In contrast, when administered with Nrf2 inhibitor ATRA, the protective effect of SR9009 was eliminated. Data are expressed as mean ± SEM, *n* = 4-5. ^*^ *p* < 0.05, ^**^ *p* < 0.01.

To investigate the influence of SR9009 on the cerebral infarction in mice with MCAO, TTC staining was performed at 24 h after MCAO. As indicated in Figure 3, SR9009 treatment remarkedly decreased the ischemia-induced infarct volume (*p* < 0.01) and ATRA increased the infarct volume (*p* < 0.01). Nevertheless, ATRA combined with SR9009 treatment increased the infarct volume compared with SR9009 group (*p* < 0.05). This suggests that protection of SR9009 against ischemic stroke injury is blunted with ATRA.

### SR9009 reduces inflammatory and oxidative damage following ischemic stroke

**FIGURE 4:**
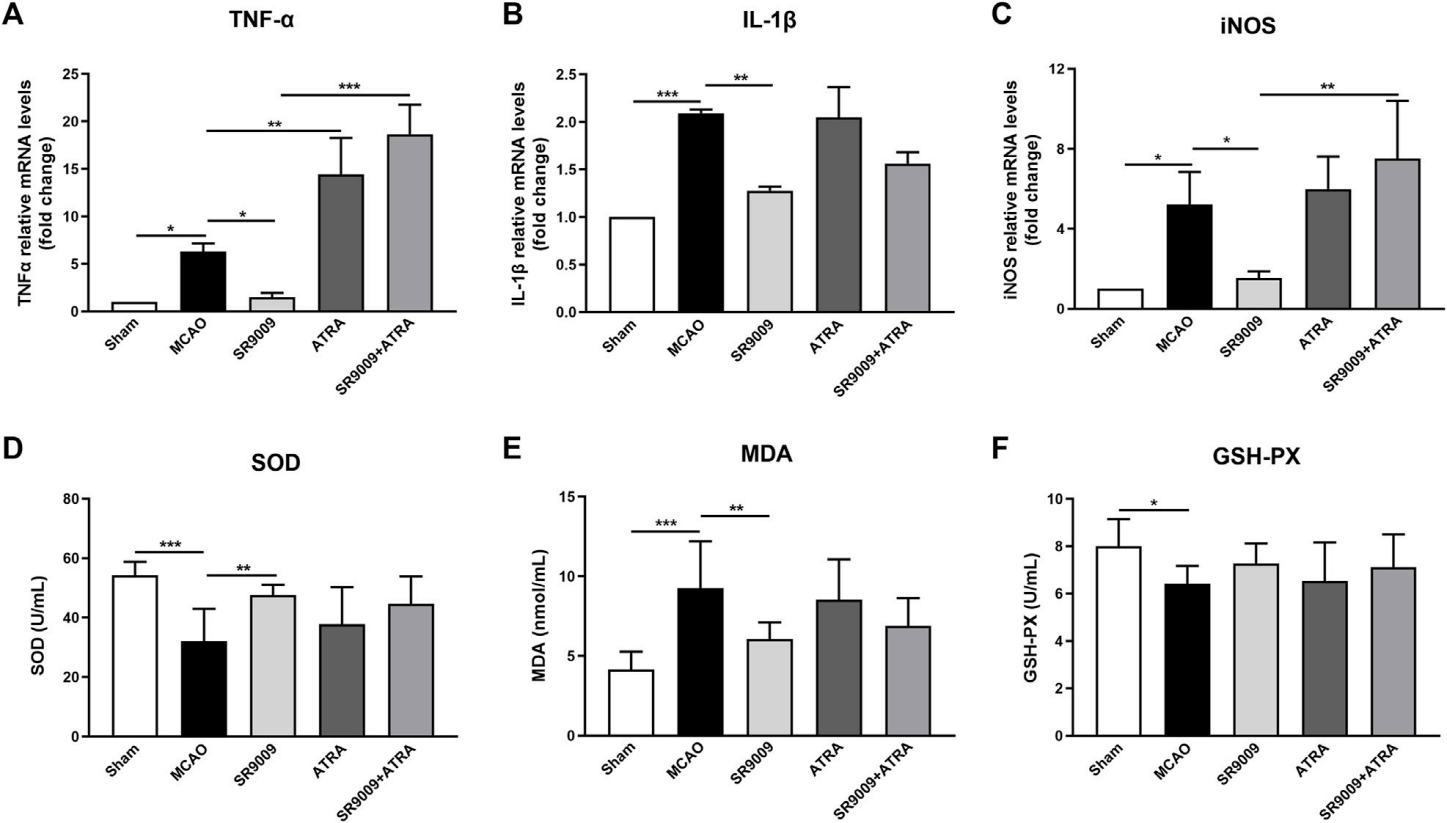
SR9009 reduces ischemia-induced inflammatory and oxidative damage in a Nrf2-dependent manner. The effect of SR9009 on neuroinflammation following ischemia was evaluated by measuring the mRNA levels of inflammatory mediators TNFα, IL-1β and iNOS in the ischemic cortex at 24 h after MCAO **(A–C)**. Compared to Sham controls, ischemic injury evoked prominent upregulation in above markers. SR9009 significantly downregulated the expression of pro-inflammatory mediator TNFα, IL-1β and iNOS after MCAO. In contrast, Nrf2 inhibitor exacerbated the neuroinflammation induced by ischemic injury indicated by the increase of TNFα, IL-1β and iNOS at mRNA levels. ATRA reduced the protective effect of SR9009 on inflammation. The oxidative parameters SOD activity, MDA content and GSH-PX activity in serum **(D–F)**. Similar effects were also shown in the oxidative parameters. Data are expressed as mean ± SEM, *n* = 8. ^*^ *p* < 0.05, ^**^ *p* < 0.01, ^***^ *p* < 0.001.

To evaluate whether SR9009 protects against the inflammatory and oxidative damage in the ischemic stroke, multiple inflammatory mediators including TNFα, IL-1β and iNOS (Figures 4A–C) in the ischemic cortex were detected at 24 h after MCAO. It is showed that the mRNA expression levels of TNFα, IL-1β and iNOS in the MCAO model group were upregulated, which was significantly reduced by SR9009. Particularly, when administered with the Nrf2 inhibitor ATRA, SR9009 did not attenuate the expression levels of the markers above. Together, we measured SOD, GSH-PX activity and MDA levels (Figures 4D–F) in serum to determine whether SR9009 attenuated oxidative damage in mice induced to ischemic injury. SOD and GSH-PX activity levels were decreased in serum (*p* < 0.05), whereas MDA content increased (*p* < 0.05), when compared with levels in the Sham group. SR9009-treated mice displayed significantly higher activity of SOD (*p* < 0.01) and lower serum concentration of MDA (*p* < 0.01), than did vehicle-treated mice.

### SR9009 enhances Nrf2 and downstream cytoprotective proteins levels

**FIGURE 5:**
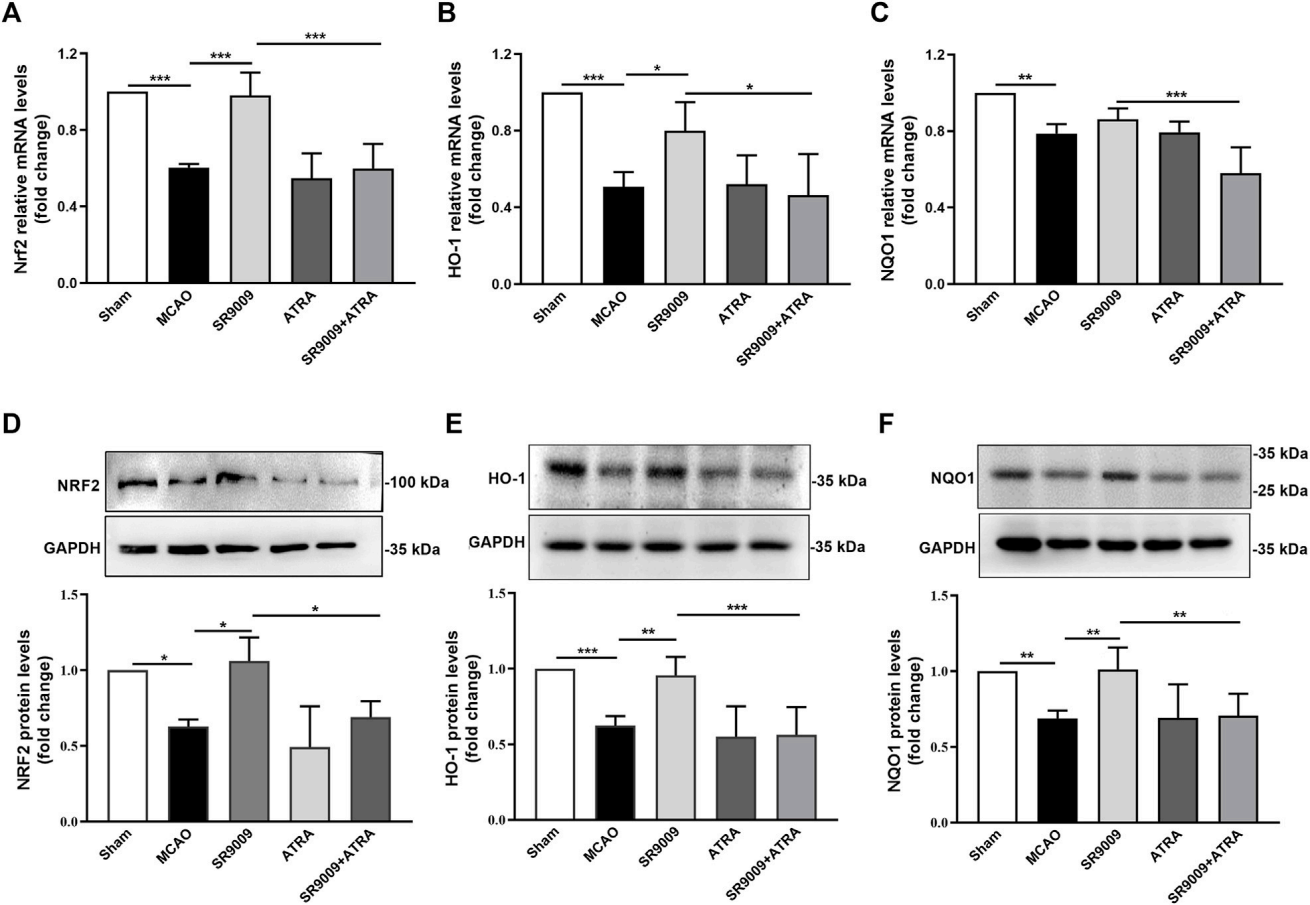
SR9009 improves the expression levels of Nrf2 and its target cytoprotective proteins after ischemia. The mRNA of Nrf2, HO-1 and NQO1 in the ischemic cerebral cortex 24 h after MCAO **(A–C)**. Data are expressed as mean ± SEM, *n* = 3. The protein of Nrf2, HO-1 and NQO1 in the ischemic cerebral cortex 24 h after MCAO **(D–F)**. Data are expressed as mean ± SEM, *n* = 6. ^*^ *p* < 0.05, ^**^ *p* < 0.01, ^***^ *p* < 0.001. It was shown that ischemic injury led to significant downregulation of Nrf2 and its target cytoprotective genes expression at both mRNA and protein levels, while SR9009 remarkably attenuated such reduction. Strikingly, the SR9009 effect was abolished when administered with ATRA.

To evaluate whether the underlying mechanisms of SR9009 involves Nrf2 pathway, the mRNA (Figures 5A–C) and protein (Figures 5D–F) levels of Nrf2 and its target genes HO-1 and NQO1 in the ischemic cortex were measured at 24 h after MCAO. Indeed, qPCR analysis indicated that, compared with MCAO controls, SR9009 treatment significantly increased the mRNA and protein levels of Nrf2, HO-1 and NQO1. Not surprisingly, no significant difference was detected in the expression of gene and protein levels above between the ATRA and MCAO groups (*p* > 0.05). The Nrf2 inhibition by ATRA significantly abolished the effect of SR9009 on the markers above, indicating the possible involvement of Nrf2 in the protective mechanisms of SR9009. Together, these findings suggest the underlying neuroprotective mechanisms might involve the activation of Nrf2 pathway.

### SR9009 restores circadian genes expression in the ischemic cerebral cortex of mice

**FIGURE 6:**
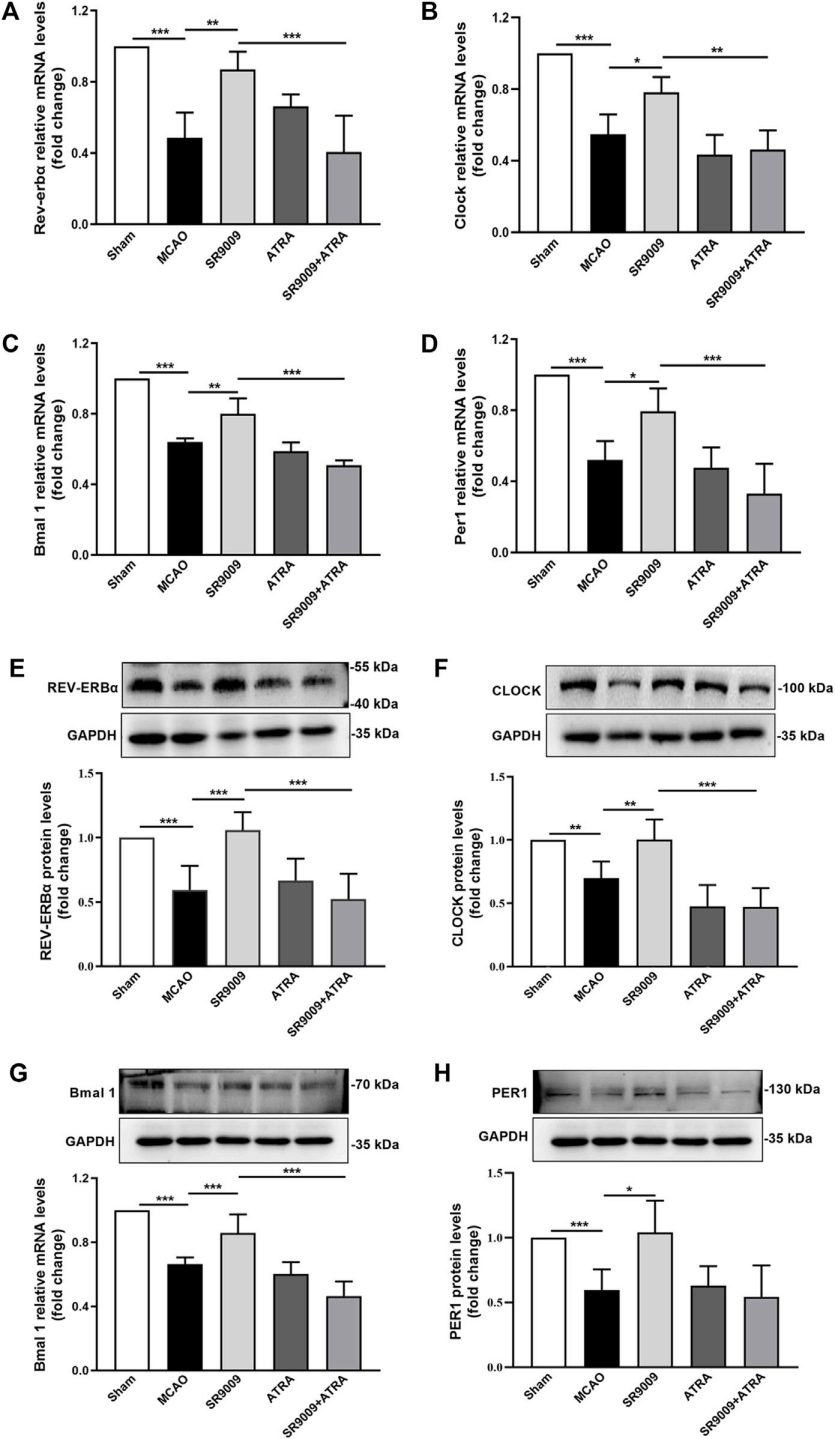
The effects of SR9009 on circadian gene and component protein expressions in the ischemic cortex. The mRNA of Rev-erbα, Clock, Bmal 1 and Per1 in the ischemic cerebral cortex 24 h after MCAO **(A–D)**. Data are expressed as mean ± SEM, *n* = 3. The protein of Rev-erbα, Clock, Bmal1 and Per1 in the ischemic cerebral cortex 24 h after MCAO **(E–H)**. Data are expressed as mean ± SEM, *n* = 6. ^*^ *p* < 0.05, ^**^ *p* < 0.01, ^***^ *p* < 0.001. It was shown that ischemic injury led to significant downregulation of circadian genes expression at both mRNA and protein levels, while SR9009 remarkably attenuated such reduction. Strikingly, the SR9009 effects were partly abolished when administered with ATRA.

To confirm the expression levels of circadian clock genes during ischemic stroke and determine whether SR9009 affects circadian clock in the ischemic cortex of mice, the gene (Figures 6A–D) and protein (Figures 6E–H) expression levels of Rev-erbα, Bmal1, Clock and Per1 were investigated at 24 h after MCAO. Compared to Sham control group, MCAO group showed a marked reduction in the mRNA and protein levels of Rev-erbα, Bmal 1, Clock and Per1. SR9009 treatment significantly attenuated the decline in these markers following ischemia, while these effects of SR9009 were dramatically abolished when administered with Nrf2 inhibitor ATRA. Meanwhile, no significant difference was observed in these markers between MCAO and ATRA groups. Together, these results indicated that ischemia led to decline of Rev-erbα, Bmal1, Clock and Per1 expression levels, while SR9009 essentially attenuated such alteration.

## Discussion

In the present study, we found the neuroprotective effects of SR9009 in a stroke model and provided a novel mechanism. SR9009 could improve nervous deficit symptoms of mice with the MCAO and reduce the brain infarct volume. It needs to be further revealed that SR9009 did not improve the rearing movement, which is a parameter of motor behavior related to exploratory activity (Thiel et al., 1999; Crusio, 2001). In addition, SR9009 could inhibit the overproduction of inflammatory cytokines, increase SOD and GSH-PX activities levels and reduce MDA levels in serum of mice exposed to MCAO. SR9009 shows excellent antioxidant activities *via* up-regulating the antioxidant gene expression of Nrf2 and HO-1. Administration of SR9009 could restore the expression of circadian genes in cerebral cortex of mice.

Rev-erbα is one of the attractive targets in the neurological disorder field in recent years. It is widely expressed in the organs and tissues and is closely associated to inflammation (Griffin et al., 2019), metabolism (Ghoshal et al., 2018; Yuan et al., 2019), tumor (Sulli et al., 2018) and Alzheimer’s disease (Roby et al., 2019; Lee et al., 2020). Its direct targeted genes except *Bmal1* and *Clock* (Crumbley and Burris, 2011), include transcription repressors e4bp4 (Zhang et al., 2021), NPAS2 (Crumbley et al., 2010), NLRP3 (Pourcet et al., 2018; Wang et al., 2018), etc. SR9009, an agonist of Rev-erbα, is highly fat-soluble and is able to penetrate the blood-brain barrier (Wang et al., 2020). It plays an agonistic effect by increasing the recruitment of recruiting co-repressors nuclear receptor co-repressor 1 (NCOR1) and histone deacetylase 3 (HDAC3) (Wang et al., 2020). The circadian rhythm of mice and the expression of circadian genes can be disrupted by the occurrence of ischemic stroke (Beker et al., 2018), including *Rev-erbα*, *Per1*, *Clock* and *Bmal1*. In our study, we found that cerebral ischemia induced decreased expression of circadian genes, including Rev-erbα.

Ischemic stroke onset occurs more frequently in the first 2 h in the morning (Peter-Derex and Derex, 2019), which was reported to have a significant influence on the infarct size (Vinall et al., 2000). Our preliminary data showed that the most severe brain infarct size was observed at ZT0 while the minimized sized was found at ZT18. This phenomenon may be related to circadian fluctuations in sleep/wakefulness (Chellappa et al., 2019), heart rate (Kodama et al., 2018), blood pressure (Gąsecki et al., 2020), platelet aggregation (Kubota et al., 1987). Therefore, ZT0 is chosen for MCAO operation in our study. Considering that the peak of Rev-erbα protein expression is ZT6 (i.e. 12:00) in the cortex and hippocampus brain regions (Zhang et al., 2021), ZT6 was chosen for the SR9009 administration in this study (Amador et al., 2016). We validated that SR9009 essentially activated Rev-erbα. SR9009 could ameliorate brain infarction and improve nerve function. Pharmacological activation of Rev-erbα mitigate damages following ischemia reperfusion injury, including reduction of cerebral infarct volume and prevention of neurological injury. Circadian genes are closely related to cardiovascular and cerebrovascular diseases. Rev-erbα gene knockout mice displayed progressive dilated cardiomyopathy and lethal heart failure (Song et al., 2022). Mice with endothelial cell-specific Bmal1 gene knockout lose sensitivity to the circadian changes of thrombotic vascular obstruction (Bhatwadekar et al., 2017). Per1 gene knockout mice are more prone to neuronal cell death after ischemic injury in hippocampus (Wiebking et al., 2013). The mean arterial pressure and heart rate circadian phase of clock mutant mice were significantly delayed (Alibhai et al., 2014). Therefore, protection against the decline of circadian genes may also be a therapeutic direction in cerebral ischemic injury.

Nrf2 has a powerful anti-inflammatory activity mediated *via* modulating NF-κB (Hassanein et al., 2020) and NLRP3 (Lin et al., 2020b). We found SR9009 mitigated the excessive release of the cytokines TNF-α, IL-1β and iNOS, while ATRA blunted the effect, suggesting a link between Nrf2 and inflammation. Nrf2 nuclear translocation augments expression of antioxidant-response elements HO-1 and NQO1. HO-1 is induced by a variety of conditions associated with oxidative stress. After stroke, the oxidative stress plays a key role in the pathophysiology of the ischemic brain (Orellana-Urzúa et al., 2020). We used ATRA, which inhibit Nrf2 activation *via* activating the RARα-Nrf2 complex in the present study (Alvi et al., 2022; Wang L et al., 2022). The protective effect of SR9009 decreased when administered with ATRA. Manifested in inflexible behavior and increased cerebral infarction area, suggesting that Nrf2 pathway contributes to protective effects of SR9009 against ischemic injury.

Yang et al. (2013). Investigated that inflammatory stimulation attenuates Rev-erbα promoter activity and expression. It is speculated that inflammation driven by ischemia reperfusion may attack the circadian clock, because inflammatory cytokines (such as TNF-α and IL-1β) are modifications of circadian genes, such as Bmal1 (Yoshida et al., 2018) and Per2 (Cavadini et al., 2007; Yoshida et al., 2013). In addition, the experimental results showed that the application of SR9009 promoted the normal expression of Per1, Bmal1, Clock and Rev-erbα in ischemic cortex. SR9009 may have an effect on the expression of Bmal1 and Clock gene and protein in cerebral ischemia mice independent of Rev-erbα. For example, Wu *et al.* revealed there is a crosstalk between key hypoxia signaling molecule HIF1A and Bmal1 (Wu et al., 2016). Thus, we may use Rev-erbα knockout mice to reveal the role of Rev-erbα in ischemic stroke in further study. Rev-erbα is generally characterized as being unable to activate downstream genes without the activated functional region 2 (AF2) region, which is necessary for transcriptional activation. The Rev-erbα is constitutive repressor of transcription owing to recruitment of transcriptional co-repressor proteins (Kojetin and Burris, 2014; Zhang et al., 2021). Therefore, Rev-erbα may achieve the positive regulation of Nrf2 through other transcription repressors, such as retinoic X receptor alpha (RXRα), which specifically inhibits Nrf2 activity (Wang et al., 2013). In addition, the regulatory mechanism of the ischemic attack controlled by the biological clock is still unclear and how the cerebral ischemia regulate Rev-erbα remains largely unresolved.

Further study is needed to demonstrate the involvement of Rev-erbα in the protective effects of cerebral ischemia by Rev-erbα deficient mice and focus on verifying the relationship between Rev-erbα and Nrf2, particularly through the use of Rev-erbα knockout mice. SR9009 is a dual Rev-erb agonist (Rev-erbα and Rev-erbβ) and we cannot totally rule out effects on Rev-erbβ receptor. Nevertheless, both nuclear receptors have overlapping functions, thereby sharing many same target genes. Moreover, other pathways involved in this process and future studies might address this possibility and the activity of Nrf2 and Keap1 remain to be determined (Bellezza et al., 2018; He et al., 2020). These results will provide a better understanding of the role of Rev-erbα and promote the development of drugs that target ligand-regulated nuclear receptors in cerebral ischemia.

## Conclusion

Taken together, we presented that Rev-erbα activation by SR9009 protects against acute ischemic brain damage and attenuated inflammatory and oxidative stress, and the underlying mechanism may involve Nrf2 pathway activation. These findings contribute to our understanding of the role of Rev-erbα activation in ischemic stroke.

## Data Availability

The original contributions presented in the study are included in the article/supplementary materials, further inquiries can be directed to the corresponding authors.
